# A Barium Swallow Study Leading to an Incidental Finding on a Screening Colonoscopy

**DOI:** 10.7759/cureus.1920

**Published:** 2017-12-07

**Authors:** Patricia Guzman Rojas, Chirin Orabi, Glenn Speth

**Affiliations:** 1 Internal Medicine, UCF College of Medicine; 2 Gastroenterology, Orlando VA Medical Center

**Keywords:** barium swallow test, esophagram, appendicolith, colonoscopy

## Abstract

Barolith is a mixture of firm feces with barium sulfate, and a frequent cause of obstruction of the appendiceal lumen that can result in appendicitis. Nonetheless, some other complications like aspiration, allergic reaction, and bowel obstruction have also been reported. We present the case of a 71-year-old man with a history of amyotrophic lateral sclerosis (ALS), who came to the gastroenterology clinic complaining of intermittent loose stools and dysphagia to solids for the past months. The patient underwent a barium swallow study six days prior and was completely normal. A colonoscopy was done showing normal appearing mucosa, with a whitish foreign object found on the appendiceal orifice. Removal of the barolith was done by means of a biopsy forceps.

Our patient did not have any signs or symptoms of appendicitis prior to the procedure, and the successful removal of the barolith was achieved. Elderly patients, and patients with decreased gastrointestinal (GI) transit, could be a population at risk for barium retention/appendicitis; for this reason, more research studies should be done to assess possible preventive treatments.

## Introduction

Barium studies are diagnostic tests that are widely performed in the United States and have currently replaced several invasive procedures. There are mainly three types of studies: barium enema, small-bowel follow through, and barium swallow. Although these diagnostic tools provide an accurate evaluation of the anatomy of the gastroenterology (GI) tract, there are several complications associated with them.

We report this case to elucidate pertinent images related to a barium swallow test, and we provide pertinent literature research about the post-procedure complications.

## Case presentation

We present the case of a 71-year-old man with a history of amyotrophic lateral sclerosis (ALS), who came to the gastroenterology clinic as a follow-up visit, complaining of intermittent loose stools and dysphagia to solids for the past months. At the initial encounter, the patient also reported associated periumbilical pain and seven to eight loose stools for the past five weeks; for this reason, he was treated with dicyclomine and loperamide. Furthermore, a course of ciprofloxacin and metronidazole was given due to a computed tomography (CT) abdomen positive for mild colitis. The patient reported persistent symptoms, despite the above-mentioned treatment. Also, he complained of dysphagia to solids and liquids and felt that it was likely related to his ALS. His primary care doctor treated him with trimethoprim/sulfamethoxazole 160 mg, which improved his diarrhea.

**Figure 1:**
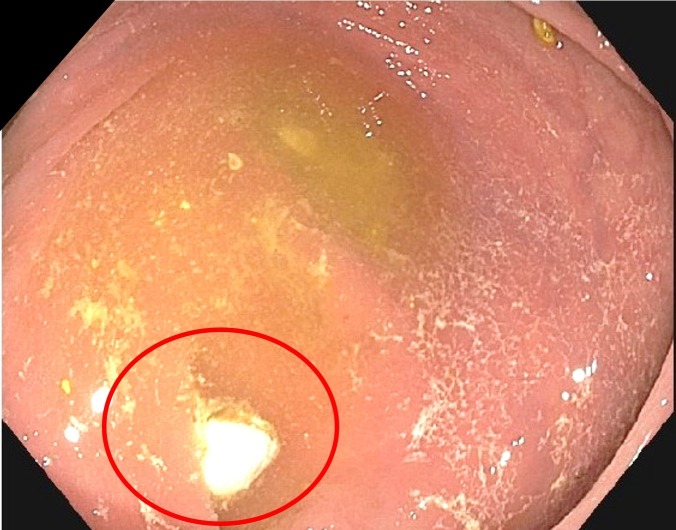
Colonoscopy: barolith at appendiceal orifice

**Figure 2:**
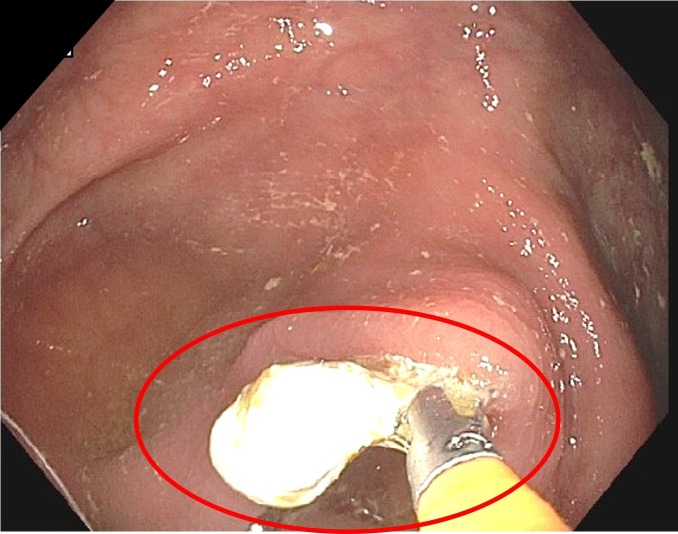
Colonoscopy: extracting barolith

**Figure 3:**
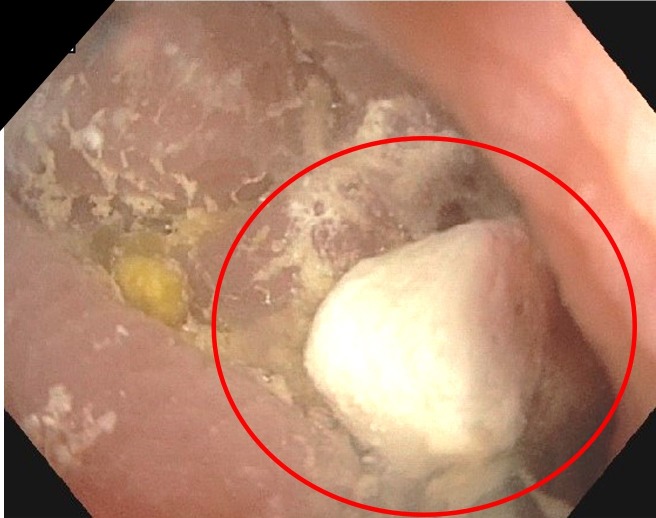
Colonoscopy: extracted barolith

The patient denied any prior screening colonoscopy and, due to the mentioned symptoms, he was scheduled for a barium esophagram, an upper endoscopy, and a colonoscopy. The barium esophagram was done six days before the endoscopy/colonoscopy, showing no abnormalities. The upper endoscopy showed erosive esophagitis, and the colonoscopy showed mild diverticulosis of the sigmoid colon; however, once the endoscope advanced to the cecum, a whitish foreign object was found in the appendiceal orifice (Figure 1). Removal of the barolith was done by means of a biopsy forceps (Figure 2). Finally, the patient was sent home with sucralfate for his esophagitis. Moreover, there weren't any reported complications after the procedure (Figure 3).

## Discussion

Appendicolith is a calcified mixture of feces with mineral deposits. Barolith is a type of appendicolith that is composed of barium sulfate. Baroliths can cause several adverse effects, including appendicitis, aspiration, allergic reaction, and bowel obstructions.

Barium obstruction can present with typical symptoms of small bowel obstruction, like abdominal pain, distention, nausea, emesis, constipation, or obstipation. Kurer, et al. [1] performed a systematic review of 31 patients with a diagnosis of barium obstruction, and they found that half of them developed symptoms more than five weeks after the barium study was done. Treatment comprised conservative measures, like laxatives; however, surgery and endoscopic dissolution were alternative options when the previous measures failed.

Barium appendicitis was first described in 1954, and it is known that approximately more than 90% of patients undergoing a barium study evacuate the barium within 72 hours [2]. For this reason, there is only a small percentage of patients who present with appendicitis after those imaging studies. It is also important to mention that the timeframe between the study and the onset of appendicitis could range from four hours up to several years [3-4].

Katagiri, et al. [5] conducted a retrospective review from the years 2013 to 2015, and found that 396 patients were admitted with the diagnosis of acute appendicits; however, only 12 of these met the definition for barium appendicitis. They concluded that an appropriate measurement of CT scan radiodensity of the material in the appendix could differentiate barium appendicitis from standard appendicitis.

Moreover, Li, et al. [6] performed a nationwide study in Taiwan, where they found a 1.19 incidence rate of appendicitis in patients who underwent a barium study. Additionally, they concluded that there is an increased, time-dependent risk of appendicitis in patients who undergo a barium study.

## Conclusions

Our patient did not have any signs or symptoms of appendicitis prior to the procedure, and a successful removal of the barolith was achieved. Elderly patients and patients with decreased gastrointestinal transit could be a population at risk for barium retention/appendicitis; for this reason, more research studies should be done to assess for possible preventive treatments. The purpose of this case report is to increase awareness of potential gastrointestinal complications after barium studies and increase prompt diagnosis.
